# Pulmonary vascular system: A vulnerable target for COVID‐19

**DOI:** 10.1002/mco2.94

**Published:** 2021-10-17

**Authors:** Jiayuan Ai, Weiqi Hong, Min Wu, Xiawei Wei

**Affiliations:** ^1^ Laboratory of Aging Research and Cancer Drug Target State Key Laboratory of Biotherapy National Clinical Research Center for Geriatrics West China Hospital Sichuan University Chengdu Sichuan PR China; ^2^ Department of Biomedical Sciences School of Medicine and Health Sciences University of North Dakota Grand Forks North Dakota USA

**Keywords:** COVID‐19, inflammation, pulmonary vascular system, SARS‐CoV‐2, thrombosis, vascular endothelial damage

## Abstract

The number of coronavirus disease 2019 (COVID‐19) cases has been increasing significantly, and the disease has evolved into a global pandemic, posing an unprecedented challenge to the healthcare community. Angiotensin‐converting enzyme 2, the binding and entry receptor of severe acute respiratory syndrome coronavirus 2 (SARS‐CoV‐2) in hosts, is also expressed on pulmonary vascular endothelium; thus, pulmonary vasculature is a potential target in COVID‐19. Indeed, pulmonary vascular thickening is observed by early clinical imaging, implying a tropism of SARS‐CoV‐2 for pulmonary vasculature. Recent studies reported that COVID‐19 is associated with vascular endothelial damage and dysfunction along with inflammation, coagulopathy, and microthrombosis; all of these pathologic changes are the hallmarks of pulmonary vascular diseases. Notwithstanding the not fully elucidated effects of COVID‐19 on pulmonary vasculature, the vascular endotheliopathy that occurs after infection is attributed to direct infection and indirect damage mainly caused by renin‐angiotensin‐aldosterone system imbalance, coagulation cascade, oxidative stress, immune dysregulation, and intussusceptive angiogenesis. Degradation of endothelial glycocalyx exposes endothelial cell (EC) surface receptors to the vascular lumen, which renders pulmonary ECs more susceptible to SARS‐CoV‐2 infection. The present article reviews the potential pulmonary vascular pathophysiology and clinical presentations in COVID‐19 to provide a basis for clinicians and scientists, providing insights into the development of therapeutic strategies targeting pulmonary vasculature.

## Graphical abstract

SARS‐CoV‐2 infection‐induced pulmonary vascular injury and potential treatments target the pulmonary vascular system.

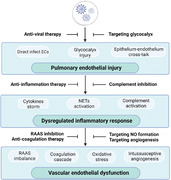

## 1 INTRODUCTION

Severe acute respiratory syndrome coronavirus 2 (SARS‐CoV‐2) is a novel beta‐coronavirus sharing 79% genome sequence identity with SARS‐CoV and 50% with Middle East respiratory syndrome coronavirus (MERS‐CoV),^1^ which causes pulmonary infections ranging from mild to severe named as coronavirus disease 2019 (COVID‐19). The increase in the number of patients suffering from COVID‐19 is exponential since its initial description and is currently a global pandemic. As of 17 September 2021, there were over 200 million confirmed infections and more than 4.5 million deaths globally.^2^ Currently, the pathogenesis of COVID‐19 is still in research state,^3^ and virus undergoes fast mutation for better survival in the human body.^4^

Pulmonary vascular homeostasis is essential for the maintenance of our normal life activities. With the exception of gas exchange, the pulmonary vasculature plays a role in carrying blood and important materials to tissues and organs and is implicated in the elimination of waste and by‐products from tissues.^5^ The endothelium of pulmonary vessels forms a barrier to prevent injury. The initial damage may be triggered by a viral infection, oxidative stress, and circulating components that occur during tissue injury and are associated with the release of histones, chemokines, cytokines, and damage‐associated proteins.^5^ These components ultimately lead to further endothelial damage and dysfunction.

The pathogen, SARS‐CoV‐2, enters the host through interaction with angiotensin‐converting enzyme 2 (ACE2) receptor expressed in pulmonary endothelium as well^6^, ^7^; thus, pulmonary vasculature is a possible target in COVID‐19. Notably, SARS‐CoV‐2 particles have been observed on vessels.^8^ Cytoplasmic vacuolization in vascular endothelial cells (ECs), capillary congestion, thrombosis, and microangiopathy in small vessels and capillaries have been observed in lungs by several post‐mortem studies.^9^, ^10^, ^11^ Further, pulmonary vascular abnormalities have been observed by chest computed tomography (CT).^12^ Indeed, vascular thickening is very common in COVID‐19, which is not present in non‐COVID‐19 pneumonia.^13^ These imaging features and clinical observations imply a potential tropism of SARS‐CoV‐2 for pulmonary vasculature and indicate that pulmonary vascular disease is a feature of COVID‐19.

Key mechanisms implicated in the pathophysiology of pulmonary vascular damage secondary to SARS‐CoV‐2 infection may include direct viral toxicity,^14^ endothelial glycocalyx (eGC) injury,^15^ ECs dysfunction associated with an imbalance of renin‐angiotensin‐aldosterone system (RAAS),^16^ coagulation cascade,^14^ oxidative stress,^14^ dysregulation of the immune response,^16^ and angiogenesis.^17^ The current review explores the possible pathogenic mechanisms and evidence of pulmonary vascular damage in COVID‐19. Further, we also summarized potential therapies targeting pulmonary vasculature to provide a guidance for treating pulmonary vascular complications associated with COVID‐19.

## 2 PULMONARY VASCULATURE

### 2.1 Importance of pulmonary vasculature in comparison with other tissues

The vascular system is also referred to as the circulatory system or vascular tree.^5^ Similar to systemic vascular beds, the pulmonary vasculature comprises three distinct components connected in series including the arterial system, capillaries, and venous system.^18^ It can be divided into two sets of blood vessels based on the source and function. Functional vessels of the lung (minor circulation) include the pulmonary arteries and pulmonary veins, which are directly involved in the gaseous exchange and comprise the entire cardiac output and maintain high blood flow at low intravascular arterial pressure.^19^ Nutrient vessels of the lung (the great circulation) include bronchial arteries and veins that systematically provide oxygenated blood to the walls of the conducting airways, pulmonary arteries, and pulmonary veins.^19^ Capillaries are key components of the systemic circulation, and pulmonary abnormalities are primarily restricted to alveolar capillaries.^20^ Notably, pulmonary vasculature differs from systemic circulation structurally and functionally in which pulmonary arteries have thinner walls with significantly less vascular smooth muscle and have no basal tone and carry mixed venous blood.^19^ In addition, alveolar epithelial cells are associated with pulmonary capillaries, potentially providing conditions for the interference of alveolar and pulmonary vascular dysfunction in COVID‐19.

### 2.2 Vascular ECs

ECs protected by pericytes line the inner layer of blood vessels and participate in supporting vessel structure. They can produce and secrete various endothelium‐derived diastole factors, such as nitric oxide (NO) and angiotensin II (Ang II), which are implicated in the regulation of vascular tone.^21^ Activated ECs release chemokines, cytokines, and adhesion molecules that augment permeability of blood vessels, and this can be suppressed by NO in resting state.^21^, ^22^ Abnormal vasoconstriction, thrombosis, and cytokines storm may occur and further aggravate endothelial injury and dysfunction. The structure and function of ECs vary among different tissues and organs.^23^ Vascular ECs are arranged in a dense monolayer, forming a protective lining that directly contacts with circulating blood components and tissue.^3^, ^24^ Therefore, it provides an interface for the exchange of gases between alveolar and red blood cells within pulmonary capillaries and facilitates basic substrate delivery. Collectively, the pulmonary vascular endothelium is a crucial orchestrator in the pathogenesis of various lung diseases.

### 2.3 Vascular eGC

**FIGURE 1:**
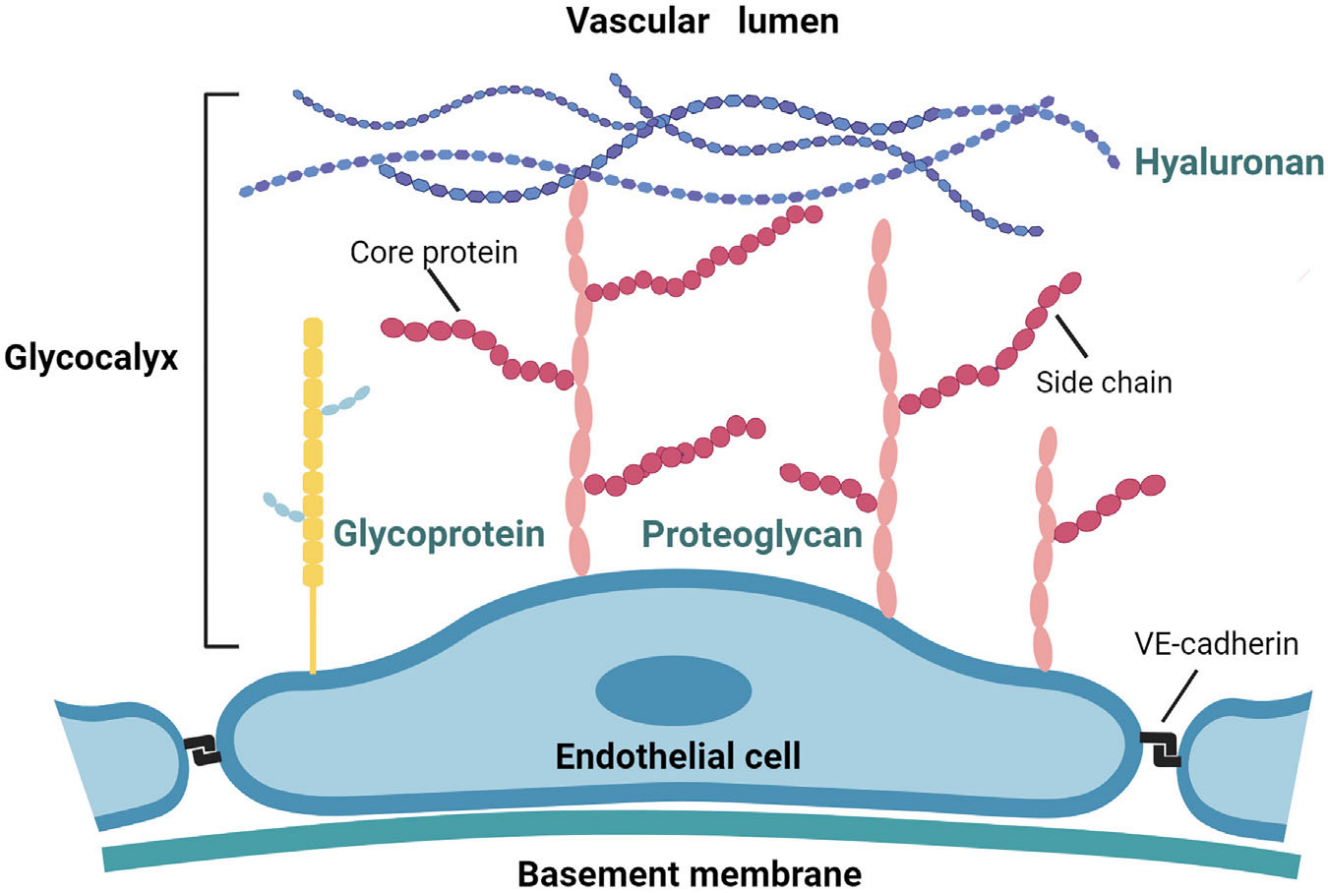
Schema of the vascular endothelium. The luminal surface of the vascular endothelial cell (EC) is covered by the glycocalyx layer, consisting of glycoproteins, proteoglycans proteins, and hyaluronan, of which glycoproteins and proteoglycans form the bulk of the glycocalyx. Proteoglycans have a protein core to which are attached negatively charged glycosaminoglycan side chains. ECs are connected by VE‐cadherin

The luminal surface of blood vessels is coated with a thin (～500 nm) gel‐like glycocalyx layer, comprising plasma proteins, glycoproteins, proteoglycans, and their associated glycosaminoglycans (GAGs) side chains, and hyaluronan^25^ (Figure 1). eGC, as the gatekeeper of vascular homeostasis, plays important roles in maintenance of endothelial permeability, integrity, and regulation of inflammatory responses and microcirculatory flow through a direct interaction with the blood.^5^, ^25^, ^26^ Specifically, eGC provides an antithrombotic surface by binding antithrombin to heparan sulfate, a major element of the glycocalyx, for regulating vascular blood flow.^27^ The GAGs side chains have negative charges as a force pulling albumin to the lumen through electrostatic repulsion.^28^ The morphology of the eGC is diverse and varies among different organs. For example, the eGC is relatively thinner in pulmonary capillaries,^29^ which is suitable for gas exchange with the alveoli and the low intravascular pressure in pulmonary circulation.^30^ Nevertheless, the pulmonary thinner eGC makes the pulmonary vasculature more vulnerable to injury.

### 2.4 Factors that affect vascular development

Several factors contribute to the regulation of the pulmonary vasculature, including transcription factors and growth factors.^19^ Vascular endothelial growth factor (VEGF) is one of the most widely studied growth factors that acts in maintaining vascular integrity through receptor/ligand interaction with VEGF‐R1 and VEGF‐R2, which control vascular organization and angiogenesis, respectively.^19^ In addition, angiopoietins (Ang 1 and 2) are essential in vascular development.^19^ Tie2 is a receptor highly enriched in endothelium and actively signals vascular quiescence.^31^ Ang1 binds to the Tie2 receptor thus exerting various effects on vascular development, including anti‐inflammatory response by inhibiting the nuclear factor kappa B (NF‐κB) signaling, maintaining vascular permeability through vascular endothelial (VE)‐cadherin, and altering the cytoskeletal morphological structure.^32^ Both Ang1 and Tie2 are expressed in ECs.^33^ Ang2 is an innate inhibitor of Ang1 and competitively antagonizes Ang1/Tie2 signaling to exert negative effects on maintaining vascular permeability. Parikh et al. deemed that excessive Ang2 is implicated in increased pulmonary vascular permeability in sepsis in humans.^34^ A previous clinical study reported that treatment of human pulmonary microvascular ECs with inflammatory mediators causes a reduction in Ang2 release, implying that the pulmonary vascular bed may not be the origin of increased Ang2 in human sepsis.^35^ Therefore, further studies should explore the role of Ang2 in pulmonary vascular complications. Furthermore, platelets play key roles in maintaining vascular integrity^36^ and exhibit endothelial barrier function, especially under inflammatory conditions.^37^ Platelets detect invading pathogens and mediate immune responses directly through interaction with neutrophils, monocytes, and lymphocytes and indirectly amplify the immune response by releasing cytokines and antimicrobial peptides.^38^ Therefore, platelets are important effectors in pulmonary immune responses.^39^

### 2.5 COVID‐19 and pulmonary blood vessels

COVID‐19 frequently attacks the lungs,^40^ causing or/and aggravating several lung diseases. The main pulmonary complications are summarized in Table 1. Numerous studies have explored endothelial dysfunction in COVID‐19 and verified that the pulmonary vascular system is implicated in SARS‐CoV‐2 infection. Chest CT scans of COVID‐19 patients suggested significant vascular thickening, compared to non‐COVID‐19 viral pneumonia.^13^ Examinations of lungs from seven patients who died from COVID‐19‐associated respiratory failure showed three unique vascular features, compared to those of influenza A (H1N1) infection. The first feature was severe endothelial damage associated with intracellular viruses and rupture of EC membranes.^17^ Moreover, pulmonary vascular histological analysis showed that COVID‐19 patients presented with extensive thrombosis with microangiopathy.^17^ Although thrombi were observed in both pulmonary and systemic circulation, alveolar‐capillary microthrombi in COVID‐19 patients were nine‐fold more frequent, compared with the number in patients with influenza (*p* < 0.001).^17^ Additionally, angiogenesis was observed and the amount of new vessel growth was 2.7‐fold higher than that in lungs from patients with H1N1 (*p* < 0.001).^17^ Although the number of samples was limited, these findings indicate a sharp difference between COVID‐19 and pathological changes in severe influenza, namely, pulmonary vascular characteristic and angiogenesis. Activation and dysfunction of pulmonary endothelium are hallmarks and the main pathological causes of acute respiratory distress syndrome (ARDS), the main cause of COVID‐19. All of these largely proves that pulmonary vasculature is involved in COVID‐19. Present evidence illustrates that SARS‐CoV‐2 infection exerts adverse effects on pulmonary vasculature by direct tropism, promoting hypercoagulative state, triggering inflammation, and even forming new blood vessels. Therefore, it is important to explore the physiological structure of the pulmonary vasculature and its microenvironment.

**TABLE 1:** Pulmonary complications of coronavirus disease 2019

**Complications**	**Incidence (%)**	**Total population**	**References**
Lung cavitation	56%	39	^150^
Venous thromboembolism	69%	26	^151^
Pulmonary embolisms	23%	26	^151^
Acute respiratory distress syndrome	56%	197	^152^
Pulmonary fibrosis	∖	∖	^7^, ^153^, ^154^
Pulmonary vasculitis or capillaritis	∖	∖	^7^
In situ pulmonary arterial thrombosis	∖	∖	^27^
Pulmonary hypertension	∖	∖	^7^
Pulmonary edema	∖	∖	^3^

## 3 PULMONARY VASCULAR PATHOLOGY IN COVID‐19

### 3.1 Vascular endothelial injury

#### 3.1.1 SARS‐CoV‐2 directly damages pulmonary ECs

Previous studies report that SARS‐CoV‐2 hijacks the cell membrane receptor ACE2 to invade the target host cell with the help of transmembrane protease serine 2; thus, pulmonary vasculature is a potential target for SARS‐CoV‐2 and ultimately initiates infection.^41^ Direct vascular damage is a plausible mechanism for COVID‐19‐associated pulmonary vascular complications. Indeed, researchers have found some outcomes to support this assumption. For example, SARS‐CoV‐2 inclusions or replication by histology and electron microscopy have been observed in vascular ECs from different organs of COVID‐19 patients,^8^, ^42^, ^43^ including the lung.^5^ In addition, autopsy samples from seven COVID‐19 patients who died from respiratory failure show the presence of intracellular virus and rupture of membranes in lung ECs, a distinctive feature for COVID‐19.^17^ Furthermore, EC apoptosis occurs in COVID‐19 patients,^44^ and proliferation of SARS‐CoV‐2 can directly trigger damage and apoptosis of ECs.^8^ The antithrombotic activity of the vascular luminal surface is significantly declined because of cellular damage and apoptosis caused by SARS‐CoV‐2 direct infecting ECs.^45^

Pericytes and ECs share a common basement membrane, which is effectively formed, maintained, and remodeled through cellular cross‐talk between these two types of cells, indicating that pericytes and ECs are highly connected.^14^ Previous studies report that ACE2 is highly enriched in pericytes of some tissues,^46^ indicating that pericytes could be potential targets for SARS‐CoV‐2. Actually, a loss or detachment of pericytes has been observed in alveolar capillaries in COVID‐19 patients, which may be implicated in promoting EC sprouting and intussusception.^47^ Therefore, ACE2‐positive vessels reported in some immunohistochemistry studies could be pericytes rather than ECs.^48^ This indicates that pericytes can potentially become a highly infectious cell population of SARS‐CoV‐2, thus disrupting the integrity of the vascular barrier, increasing vascular permeability to ultimately cause endothelial dysfunction. However, the precise role of pericytes in SARS‐CoV‐2 infection has not been fully elucidated.

These findings indicate that pulmonary vascular injury may occur due to direct viral toxicity; however, further studies should be conducted to explore ACE2 expression in ECs. Previous studies report that expression of ACE2 in ECs is significantly low or absent in vivo.^49^ In addition, human primary ECs lack ACE2 receptors at RNA and protein levels, and SARS‐CoV‐2 cannot directly infect pulmonary ECs.^50^ On the contrary, pulmonary ECs transduced with recombinant ACE2 receptors are readily infected by SARS‐CoV‐2.^50^ These findings indicate that pulmonary EC may be not a primary target for SARS‐CoV‐2 infection. More studies are supposed to further explore the specific target of SARS‐CoV‐2 to fully understand the progression of pulmonary vascular involvement. Note that ACE2 is not the only receptor for viral entry; in some cells, SARS‐CoV‐2 binds to CD147 (also known as Basigin or EMMPRIN).^51^ However, the role of CD147 and other candidate receptors for SARS‐CoV‐2 infection is still in a research state. In order to identify host cell receptors, studies analyzed genomic receptor screening almost all human membrane proteins, with SARS‐CoV‐2 capsid spike protein as the target protein, and surprisingly identified twelve receptors, including ACE2, ASGR1, and KREMEN1.^52^

#### 3.1.2 Endothelial glycocalyx injury

Vascular eGC covers the luminal surface of ECs and acts in maintaining vascular homeostasis. Disruption of eGC has been found in the early stages of critically ill patients and may be an exacerbating factor of ARDS,^30^, ^53^, ^54^ the primary cause of SARS‐CoV‐2‐triggered fatality. Notably, degradation of eGC exposes EC surface receptors to the vascular lumen, which renders ECs more vulnerable to injury and increases the risk of SARS‐CoV‐2 infection. Moreover, granulocytes and platelets adhere to ECs and result in a clot that blocks blood flow.

High concentrations of circulating antithrombin have been detected in COVID‐19 cases,^55^ implying that glycocalyx degradation destroys the binding of antithrombin and heparan sulfate, and antithrombin falls off the antithrombotic surface of glycocalyx. However, studies should further explore if this observation is related to glycocalyx degradation. Furthermore, heparan sulfate is an important component of eGC and is the main viral attachment and entry site for viruses,^56^ which further supports the hypothesis that the vascular glycocalyx appears to promote susceptibility to SARS‐CoV‐2. A previous study demonstrated sublingual microvascular glycocalyx damage in COVID‐19 patients through intravital microscopy by sidestream dark field imaging to quantify relevant indicators, including glycocalyx dimensions.^57^ These indicators may indicate the damage of pulmonary vascular glycocalyx because sublingual capillaries are partly representative of pulmonary and systemic microvasculature.^58^ Stahl et al. recently analyzed plasma and serum from 19 critically ill COVID‐19 patients, and the outcomes showed significantly increased sTie2 and syndecan‐1, indicating pathological shedding of transmembrane proteins involved in glycocalyx structure and processing.^59^ Moreover, a significantly low level of protective heparanase‐2 was observed in all patients, which was attributed to the degradation of the eGC.^59^ Although the study analyzed a limited number of samples, these findings indicate the presence of vascular glycocalyx damage in patients with COVID‐19. The glycocalyx in pulmonary vasculature is thinner implying that pulmonary vascular eGC can be easily damaged in patients with COVID‐19, making the pulmonary vascular ECs more vulnerable to SARS‐CoV‐2 and further results in a series of lethal pulmonary vascular complications. Although glycocalyx plays important roles in vascular endothelial injury, thrombosis, and vasculitis, the connection between its structure and pulmonary vasculature dysfunction in COVID‐19 has not been fully explored, and thus further studies should investigate the relationship in detail.

#### 3.1.3 The cross‐talk between epithelium and endothelium

The human body comprises 60,000 miles of blood vessels, including approximately 19 billion capillaries; thus, cells are approximately 100–200 μm from the nearest capillary under physiological conditions.^60^ The close anatomical relationship between the alveolar epithelium and pulmonary microvascular endothelium, and the assumed distribution of ACE2 on pulmonary ECs,^6^, ^61^ imply that the alveolar epithelium and pulmonary microvascular endothelium have synergistic effects following SARS‐CoV‐2 infection. The alveolus chiefly consists of the epithelium and extracellular matrix surrounded by capillaries, thus forming an alveolar‐capillary barrier that prevents pathogen invasion. Although SARS‐CoV‐2 infection is often confined to the respiratory system, clinical and pathological findings indicate impairment of alveolar‐capillary barrier in COVID‐19, causing viral leakage to the circulatory system.^62^ Notably, studies report that SARS‐CoV‐2 mainly invades alveolar epithelial cells and utilizes their cellular machinery for massive replication.^62^ Direct exposure to SARS‐CoV‐2 presents an inconspicuous effect on the pulmonary endothelium, whereas treatment with culture supernatants from infected alveolar epithelial cells results in severe injury to ECs, including excessive mitochondrial fragmentation and adhesive junction damage.^62^ This finding indicates that SARS‐CoV‐2 damages pulmonary microvascular endothelium potentially through substances released from the alveolar epithelium. SARS‐CoV‐2 infection activates antiviral and immune responses of epithelial cells and upregulates proinflammatory cytokine such as interleukin 1α (IL‐1α) and interferon, which are then released into the extracellular space further damaging adjacent endothelium.^62^

### 3.2 Vascular endothelial dysfunction

#### 3.2.1 RAAS imbalance

**FIGURE 2:**
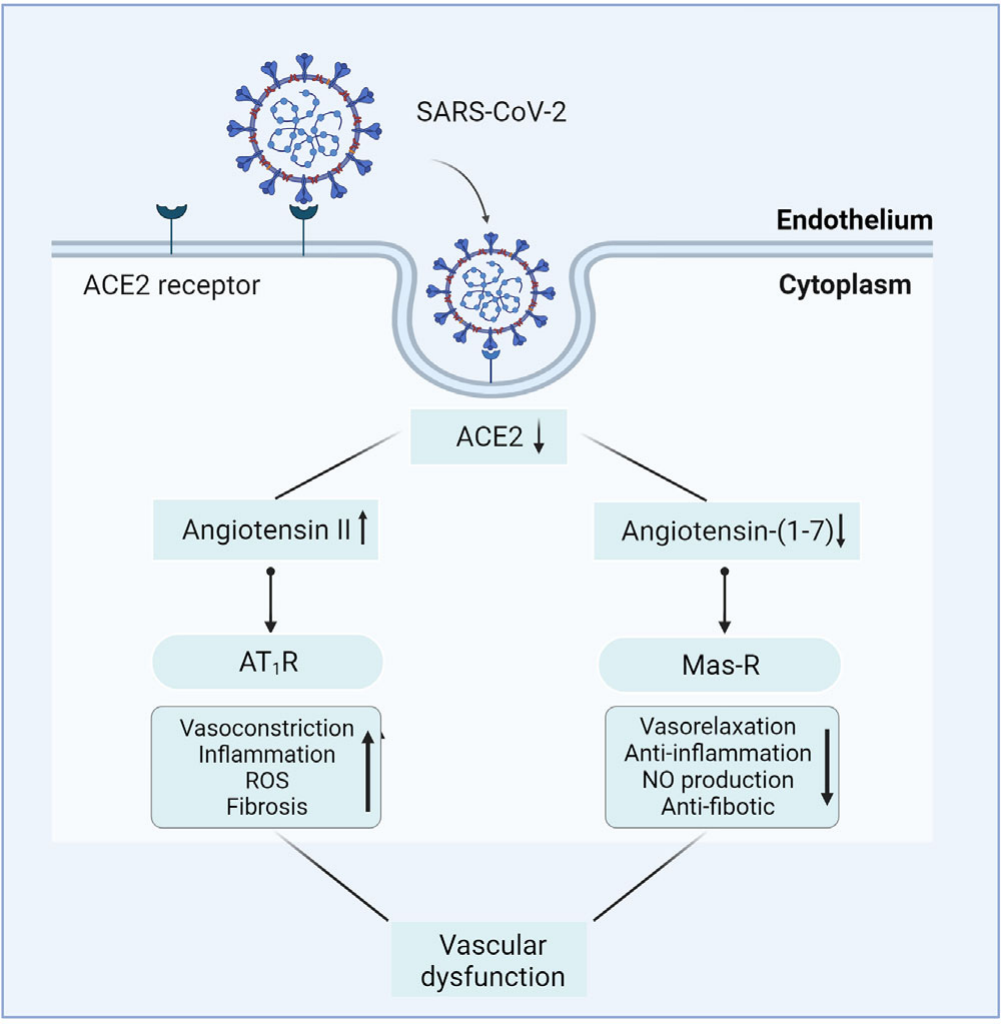
Severe acute respiratory syndrome coronavirus 2 (SARS‐CoV‐2)‐mediated angiotensin‐converting enzyme 2 (ACE2) downregulation leads to renin‐angiotensin‐aldosterone system (RAAS) imbalance and vascular dysfunction. In the setting of SARS‐CoV‐2 infection, the major subunit 1 protein (S1) of SARS‐CoV‐2 directly binds to ACE2 expressed on the endothelium. Subsequent downregulation of ACE2 activity results in an upregulation of angiotensin II (Ang II) and downregulation of angiotensins‐(1‐7), which could cause an adverse vascular consequence, such as vasoconstriction, inflammation, and fibrosis

Dysregulation of the RAAS is another possible pathophysiological mechanism of vascular endothelial dysfunction in COVID‐19. RAAS is vital for vascular homeostasis by playing various functions including regulation of plasma volume and modulation of vascular tone and inflammation.^20^ ACE2, the chief receptor for SARS‐CoV‐2 entry, has been validated on smooth muscle cells of pulmonary vessels and vascular endothelium^6^ and is a potent counter‐regulator of RAAS pathway.^63^ SARS‐CoV‐2 invades host cells by binding to ACE2. Therefore, the level of ACE2 in the host is decreased, subsequently resulting in a reduction of Ang II inactivation and the decreased conversion to angiotensin‐(1‐7; Ang‐(1‐7)). The deposition of Ang II induces vasoconstriction^64^ and regulates endothelial activation and the production of IL‐6 and reactive oxygen species (ROS) through angiotensin type 1 (AT_1_) receptor.^65^ In addition, the decreased Ang‐(1‐7) level suppresses NO synthesis, which triggers thrombus owing to leukocyte and platelet adhesion and vasoconstriction, and causes inflammation through the inflammatory cell immigration to the vessel wall^27^ (Figure 2).

Previous clinical reports show a higher level of Ang II in COVID‐19 patients, compared with healthy individuals,^66^ which is attributed to SARS‐CoV‐2 infection‐mediated ACE2 downregulation and is correlated with viral load and pulmonary injury.^67^, ^68^ Although the number of samples used in these studies was small, the findings could suggest a pathophysiological link between RAAS imbalance and COVID‐19‐related intravascular thrombotic disease in addition to the known procoagulant effects of Ang II.^20^ Notably, a recent study reported that ACE2 levels were not significantly different in the lung; however, the level was downregulated in both kidney and heart, which may be mainly attributed to ACE2's modulatory role in Ang II. The study analyzed 144 autopsy tissue samples collected from 19 COVID‐19 patients who died from SARS‐CoV‐2 pneumonia or respiratory failure.^69^ Hence, the connection between RAAS imbalance and pulmonary vascular lesions should be explored further.

#### 3.2.2 Coagulation cascade

**FIGURE 3:**
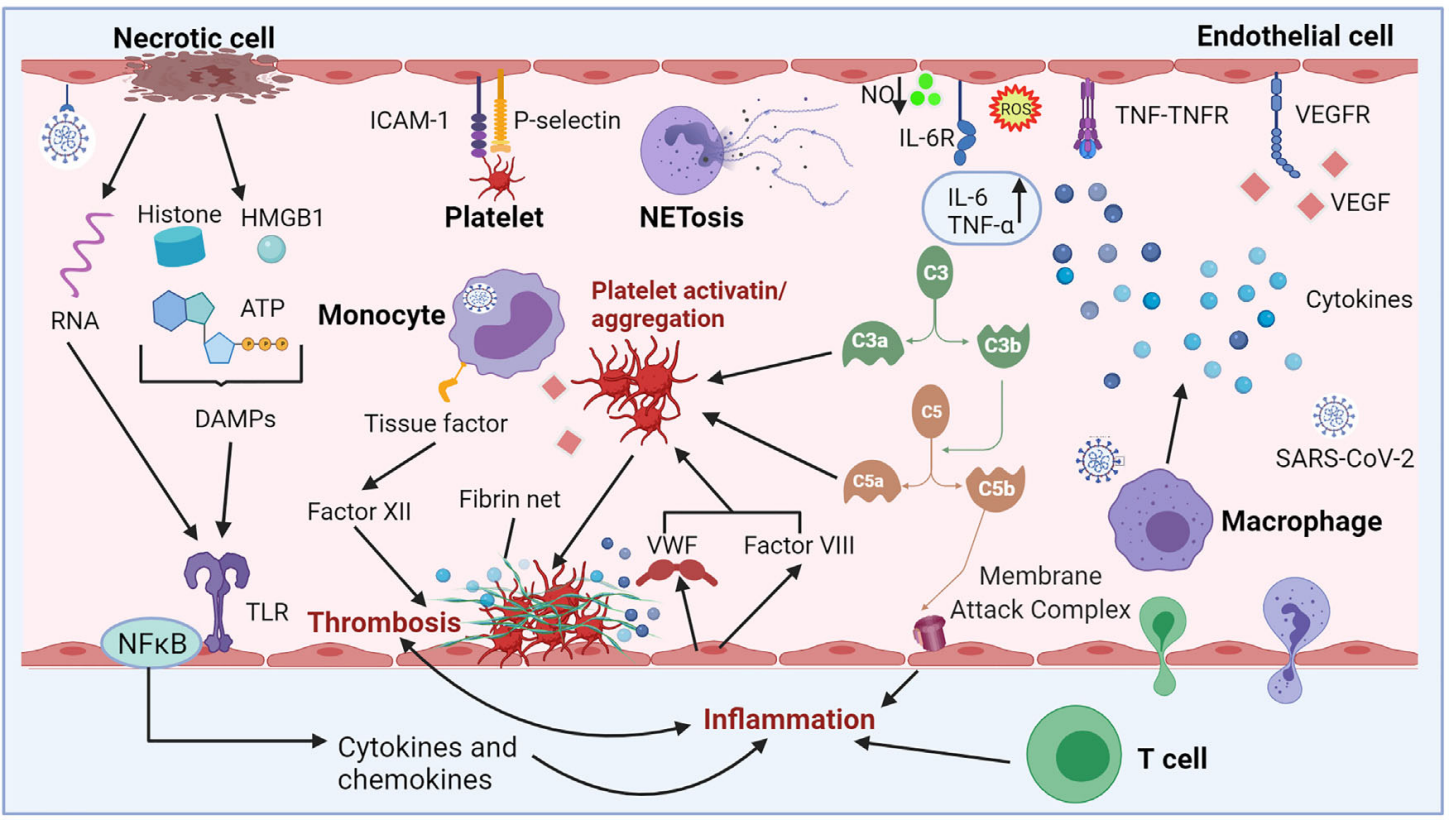
Mechanism of dysregulated inflammation and thrombosis in coronavirus disease 2019. Direct tropism of SARS‐CoV‐2 on vascular ECs leads to apoptosis/necrosis of ECs, followed by the release of intracellular cell components, such as RNA and damage‐associated molecular patterns (DAMPs; high mobility group box‐1 (HMGB1), adenosine triphosphate (ATP), and histone). These immunogenic components could bind and activate the specific toll‐like receptors, and drive the nuclear factor kappa B‐mediated transcription of pro‐inflammatory cytokines. Activated T cells release pro‐inflammatory mediators and cause direct cytotoxicity. Besides, activated monocytes present activated tissue factor and triggers coagulation cascade via activating factor XII. Neutrophils are recruited and contribute to this process through the release of neutrophil extracellular traps (NETs). Activated ECs can secrete factor VIII and von Willebrand factor from Weibel–Palade body and augment platelet production and aggregation. C3a and C5a can also facilitate this process. ICAM‐1 and P‐selectin can promote platelet to adhere to ECs, while platelet aggregation and adherence to the ECs are the primary cause of thrombosis. SARS‐CoV‐2 infection causes endothelial injury, and then excessive cytokines are released from ECs and immune cells; in particular, tumor necrosis factor α and interleukin 6 have been observed. Various cytokines are predicted to cause endothelial activation and dysfunction, leading to inflammation and high permeability of vascular. HMGB‐1, ATP, DAMPs, toll‐like receptors

Coagulation is a highly well‐coordinated process comprising the interaction of ECs, platelets, and coagulation factors. Maintenance of the dynamic balance between pro‐coagulant and fibrinolytic factors in the vascular system largely depends on vascular endothelium and its immunoregulatory function.^70^ Under the resting state, vascular endothelium acts as a mechanical barrier to prevent invasion of unwanted substrates from the blood into subcutaneous tissues, thus promoting physiological blood flow and preventing blood clotting. Stimulation of vascular endothelium by SARS‐CoV‐2 infection results in expression of tissue factor by monocytes/macrophages, thus effectively triggering clotting cascades, while the ECs secreting factor VIII and von Willebrand factor (VWF) are produced from Weibel–Palade body to augment platelet production.^27^, ^71^ Further, the released VWF stimulates platelet aggregation, and unusually large VWF prompts platelet adhesion to ECs.^27^ Platelet activation is the major cause of thrombosis and can lead to expression of VEGF, thus promoting expression of tissue factor.^71^ Moreover, excess immune effectors, such as neutrophil extracellular traps (NETs), can enhance thrombotic response.^72^ In addition, endothelial damage and dysfunction triggered by proinflammatory cytokines and tropism of SARS‐CoV‐2 for ACE2 receptors reduce the bioavailability of NO and induce venous thromboembolism and abrogate natural antithrombotic state.^73^ Changes in these prothrombotic factors result in a vicious circle that can ultimately result in microthrombi deposition and microvascular dysfunction (Figure 3).

Perturbations of the endothelium caused by SARS‐CoV‐2 infection can lead to vascular leakage and promote inflammation, ultimately predisposing vasculature to a pro‐coagulation state or leading to coagulation dysfunction. Importantly, findings from the autopsy of COVID‐19 patients showed extensive pulmonary vascular thrombosis, microvascular thromboembolism, capillary congestion, and deep vein thrombosis,^74^ which were attributed to hypercoagulability owing to dysregulation and inflammation of pulmonary vascular ECs. Endothelial tumefaction and several pulmonary megakaryocytes in lung capillaries are signals of activation of the coagulation cascade.^75^ Additionally, studies demonstrate that the level of alveolar‐capillary microthrombi was nine‐fold in people who died of COVID‐19, compared with that of patients who died of influenza.^17^ However, clinical studies should be conducted to verify these findings. Increased levels of fibrinogen, fibrin degradation products, D‐meter, VWF, and factor VIII were observed in COVID‐19 patients, which indicate the severity of the disease and thrombotic risk.^55^, ^76^, ^77^ Critically, increased levels of VWF and factor VIII are unique characteristics of coagulopathy in COVID‐19,^55^, ^78^ and vascular response to SARS‐CoV‐2 infection. For healthy individuals, thrombomodulin in circulation is considered to be generated by physiological cleavage and shedding of membrane‐bound thrombomodulin, whereas the elevated level of soluble thrombomodulin is secondary to direct EC injury under hyper‐inflammatory conditions.^79^ Interestingly, a study first demonstrated that soluble thrombomodulin, a specific marker of EC injury, is relevant to hospital discharge status and segregates with the survival in COVID‐19 patients.^76^ These pathological observations indicate that vascular ECs damage by the generation of a prothrombotic milieu is a potential pathophysiological mechanism of pulmonary vascular complications of COVID‐19.^20^, ^80^

#### 3.2.3 Oxidative stress

Oxidative stress is a systemic imbalance between the relative rates of oxidant generation and levels of antioxidants.^81^ Studies report that oxidative stress plays an imperative role in endothelial dysfunction through several mechanisms, and decreased NO bioavailability is involved in the predominant pathway.^81^ Reduced expression of endothelial NO synthase (eNOS), lack of substrates for eNOS, inactivation of eNOS, and increased degradation of NO may cause a decrease in NO bioavailability.^81^ Notably, the level of NO in serum of COVID‐19 patients is low indicating the presence of oxidative stress. After binding to ACE2, SARS‐CoV‐2 enters the host cell primarily through endocytosis. ACE2 is internalized and downregulated on ECs resulting in RAAS imbalance. Once ACE2 expression in ECs is downregulated, the production of Ang‐(1‐7) is reducing, thus lowering the release of NO from ECs and consequently leading to vasoconstriction, platelet aggregation, and destruction of cellular autoimmunity.^82^ The significance of endothelial dysfunction is that older people and patients with pre‐existing risk factors for endothelial damage have a higher risk of severe SARS‐CoV‐2 infection.^82^

Another major source of oxidative stress is ROS from mitochondria.^81^ Production of ROS is necessary for maintaining normal intravascular homeostasis. Enzymes, such as nicotinamide adenine dinucleotide phosphate (NADPH) oxidase (NOX) and distinctively expressed eNOS, are involved in ROS production in vasculature. After viral infection, activation of ECs and regulation of adhesion molecules induce activation of neutrophils, which leads to a large number of histotoxic mediators production including ROS.^83^ ROS can activate calcium and NF‐κB signaling pathways to induce adhesion molecules and pro‐inflammatory factors and then result in dysregulated antioxidant mechanisms, such as nuclear factor erythroid 2‐related factor 2 (Nrf2) and antioxidant response element signaling, which ultimately increase vascular permeability and promote leukocyte adhesion.^84^, ^85^, ^86^ Notably, Nrf2 activators are a potential therapeutic strategy for suppression of SARS‐CoV‐2 entry.^87^ An emerging study suggested that oxidative stress, triggered by NOX2 activation, is implicated in the pathogenesis of COVID‐19 and may be associated with thrombotic events observed in COVID‐19 patients.^88^ Hence, the beneficial effects of antioxidant drugs on endothelial function should be examined in the development of COVID‐19 therapy.

### 3.3 Immune dysregulation

Although the pathogenesis of pulmonary vascular dysfunction in COVID‐19 is not fully clarified, it may not be solely caused by a direct viral infection of ECs. Endocytosis or membrane fusion of SARS‐CoV‐2 to host cells can result in ECs injury or apoptosis, which activates the immune response and triggers the secretion of cytokines, thus inducing an excessively inflammatory status^89^ (Figure 3). This was confirmed in a post‐mortem sample study of three COVID‐19 patients. Varga et al. found endotheliitis (characterized by an accumulation of inflammatory cells associated with endothelium, as well as apoptotic bodies) in pulmonary samples.^8^ In addition, ECs act in regulating local and systemic inflammatory and immune responses,^90^ and they can be activated through the exaggerated inflammatory response to SARS‐CoV‐2 with a large amount of pro‐inflammatory cytokines release in an event named as “cytokine storm.”^91^ The cytokine storm may lead to loss of integrity of the vascular barrier and induction of pulmonary edema, thus causing endothelialitis and activating coagulation cascade.^14^ This presents an indirect mechanism of pulmonary endothelial dysfunction in COVID‐19. Indeed, studies have demonstrated that proinflammatory cytokines and chemokines, such as tumor necrosis factor α (TNF‐α), IL‐1β, IL‐6, and granulocyte‐colony stimulating factor are significantly upregulated in COVID‐19 patients.^92^, ^93^, ^94^ Moreover, macrophage activating syndrome, T‐cell lymphodepletion, reduction of natural killer cells, and subsequent immune exhaustion have been observed in severe COVID‐19 patients,^95^ owing to overactivation of innate immunity. The vicious cycle relating to hyper‐inflammation and microvascular endothelial injury may be a pivotal factor for multiple organ failure and even death in patients with severe COVID‐19.^96^ Therefore, it is necessary to track the incipient source of the cytokine storm responding to SARS‐CoV‐2 infection and the virological mechanisms behind it to improve the prognosis of patients.^97^

#### 3.3.1 IL‐6/ IL‐6 receptor (IL‐6R)‐mediated immune dysregulation

IL‐6 plays a crucial role in COVID‐19‐associated cytokine storm. A high level of circulating IL‐6 has been observed in COVID‐19 patients based on clinical reports, which is correlated with the pathogenesis of COVID‐19^92^ and disease severity.^98^ IL‐6 overproduction can be induced by SARS‐CoV‐2 to escape immune surveillance.^99^ Notably, IL‐6 triggers the Janus kinases/signal transducers and activators of transcription (JAK/STAT) signaling pathway through the membrane‐bound IL‐6R and in turn mediates several vascular effects, including endothelial activation and dysfunction, vascular permeability, immune cell recruitment, and vascular hypertrophy and fibrosis.^100^ Besides, IL‐6 directly reduces the synthase activity and the expression of endothelial NO, as well as increases the level of vascular superoxide, which rapidly inactivates and limits NO bioavailability.^100^ Consequently, these events can alter pulmonary vascular properties and mediate vascular injury.

#### 3.3.2 Neutrophil activation causes endothelial dysfunction

Neutrophils are effectors of vascular inflammation and can lead to the formation of NETs when activated.^72^ In addition, neutrophils can promote thrombosis and microvascular injury.^101^ A previous study reported that excessive neutrophil recruitment was implicated in the severity of COVID‐19 and led to the complications observed in some patients.^102^ This is indicated by the upregulation of key cytokines involved in NET regulation and production in SARS‐CoV‐2‐infected samples, including IL‐6, TNF‐α, and CCL20.^102^ IL‐6 and TNF‐α are potent inducers of the release of NETs (NETosis),^103^ a novel form of cell death distinct from apoptosis.^104^ Studies report that a NET is the main cause of vascular damage in COVID‐19 patients.^102^ Veras et al. demonstrated that NETs were induced by SARS‐CoV‐2, and the level of NETs was augmented in plasma and lung autopsy tissues from COVID‐19 patients.^105^ This indicates that neutrophils are active in pulmonary tissues and can reach the vasculature. Extracellular histones, the elements of NETs, are cytotoxic to ECs,^106^, ^107^ especially in lungs.^106^ This may explain why COVID‐19 patients experience microvascular injuries.^108^ Moreover, neutrophil activation and inflammatory conditions characterized by high levels of extracellular histones and cytokines can damage eGC, resulting in degradation shedding of syndecan‐1, heparan sulfate, and hyaluronan,^109^, ^110^ which exposes ECs to histones and subsequently leads to decreased vasodilation and death of ∼25% of cells.^111^

#### 3.3.3 Complement activation

The complement system is an essential arm of the earliest immune response to infections and comprises approximately 60 proteins.^112^ These protein components are inactive in a non‐disease state. Once the pathway is activated, some of the pathway proteins undergo proteolytic cleavage, resulting in the formation of active enzymes that induce further protein cleavage.^113^ The pathogen promotes local complement activation and inflammation in the early stages of infection. Complement cascades may become over‐activated or even dysregulated when the injury is intensive or persistent, resulting in cell or tissue damage and organ dysfunction. Complement activation mainly occurs through three distinct pathways: the “classical pathway,” the “mannose binding lectin (MBL) pathway” and the “alternative pathway.” Once triggered, the complement cascades produce a number of effectors, including (1) opsonins (C3b and C4b) that mark cells or foreign invaders for phagocytosis; (2) the anaphylatoxins (including C5a, C3a, and C4a), which are important immunostimulators for vascular permeability and inflammatory cell recruitment; and (3) membrane attack complex (MAC; C5b‐9) can lead to cell lysis and other forms of cellular damage.^48^ Overproduction of C5a can initiate cytokine storms leading to endothelial damage and/or acute lung injury.^114^ This indicates that activation of complement cascade makes ECs vulnerable and/or dysfunctional.

Over‐activated complement system has been proved in SARS‐CoV‐2 infection cases and is a major pathological mechanism of the pulmonary vascular disease in COVID‐19. Magro et al. found extensive deposition of alternative and MBL pathway components, including C5b‐9, C4d, and MBL‐associated serine protease (MASP)2 in pulmonary microvasculature.^115^ This significant complement activity is implicated in the activation of C5b‐9/MAC, leading to microvascular endothelial injury and activation of the coagulation pathway.^48^ In addition, researches in COVID‐19 patients indicate high plasma levels of sC5b‐9 and C5a accompanied by activation of ECs.^116^ Further, studies report that aberrant complement activation is induced in lungs through a conserved, direct interaction between MASP‐2 and coronavirus nucleocapsid (N) proteins.^117^ Pulmonary injury can be significantly alleviated both in vivo and in vitro by blocking the interaction between N protein and MASP‐2 or by inhibiting complement activation.^117^ Notably, another study reported that SARS‐CoV‐2 spike proteins (subunits 1 and 2), rather than the N protein, directly stimulates the alternative pathway of complement, serving as another mechanism for activation of complement cascades.^118^ Similarly, co‐localization of COVID‐19 spike glycoproteins with C4d and C5b‐9 has been reported in the interalveolar septa and the cutaneous microvasculature in two COVID‐19 cases explored by Magro et al.^115^ Generally, these findings suggest that complement cascade is significantly activated in lung tissue of COVID‐19 patients and results in damage of the pulmonary vascular system (Figure 3).

### 3.4 Intussusceptive angiogenesis (IA)

Higher vessel growth has been detected in the lungs of COVID‐19 patients, compared to that in patients who died of H1N1.^17^ Growth of new blood vessels can occur through either traditional sprouting angiogenesis (SA) or non‐sprouting IA. “Intussusceptive” (non‐sprouting) angiogenesis is a common morphogenetic process in cancer,^119^ inflammatory diseases, and tissue regeneration.^120^ It is a rapid process whereby intussusception pillars invaginate into the lumen and splits a single vessel into two. The process is implicated in the recruitment of bone marrow‐derived mononuclear cells that expand and adapt to the capillary plexus without active proliferation of ECs.^121^ The newly formed “intussusception pillars” are then infiltrated by pericytes and myofibroblasts to provide mechanical stability to the transcapillary pillar core.^60^ Previous studies show that the development of new pillars is limited to regions of low wall shear stress and low blood flow velocity in dilated vascular segments.^60^, ^122^ Unlike IA, SA is characterized by the formation of new ECs.

The role of the virus, as a key regulator of angiogenesis, has been widely explored. It exerts its activity through the tropism of ECs to activate blood vessels, produce chemokines and/or growth factors, induce inflammation, and create a microenvironment that promotes angiogenesis.^123^ Ackermann et al. reported that the increased level of IA may be a major mechanism of pulmonary angiogenesis in COVID‐19, even during the late stages of chronic lung injury.^17^ Furthermore, CXCL12/CXCR4 signaling pathway is thought to be essential for the regulation of angiogenesis and hypoxia.^124^ The positive feedback loop between vascular shear stress, CXCL12 (stromal cell‐derived factor‐1)‐expression, hypoxia, and release of eNOS is an adaptation of the vascular system to maintain blood flow in response to long‐term inflammation. Thus, the significantly increased release of eNOS cascade is a vital biological process to maintain blood flow into tissues with occluded vessels and to initiate tissue repair via IA expanding vascular architecture.^60^ Indeed, a high expression level of CXCL12 and CXCR4 has been detected in the lungs of COVID‐19 patients,^17^ which is probably associated with intensive T‐cell infiltration.

Moreover, fibroblast growth factor 2 (FGF2) upregulation has been observed in patients who died of COVID‐19.^121^ FGF2 plays different roles in some viral infections. For instance, upregulated FGF2 is involved in MERS‐CoV‐induced strong apoptotic response important for its high lytic replication cycle in pulmonary cells, whereas it plays a protective effect on acute lung injury induced by influenza. In addition, FGF2 is implicated in the regulation of IA, acting on pericytes (essential for the development of intraluminal pillars) and endothelium, and inflammation and hypoxia may promote FGF2‐induced angiogenesis.^121^ Besides, Ackermann et al. reported upregulated level of the gene encoding VEGF‐A in patients who died of COVID‐19 and H1N1, whereas VEGF‐C, the main effector of lymphangiogenesis, was only upregulated in COVID‐19 patients.^17^ It is worth noting that VEGF acts in IA. Further, the downregulated level of VEGF induces vascular tree regression by intussusceptive vascular pruning.^125^ This finding implies that VEGF suppression is associated with vascular normalization by modulating IA, which surprisingly provides a novel ideal for the treatment of IA in COVID‐19. Further research should be conducted to explore the relationship between these findings and the clinical course of SARS‐CoV‐2 infection.

## 4 POTENTIAL THERAPIES

**FIGURE 4:**
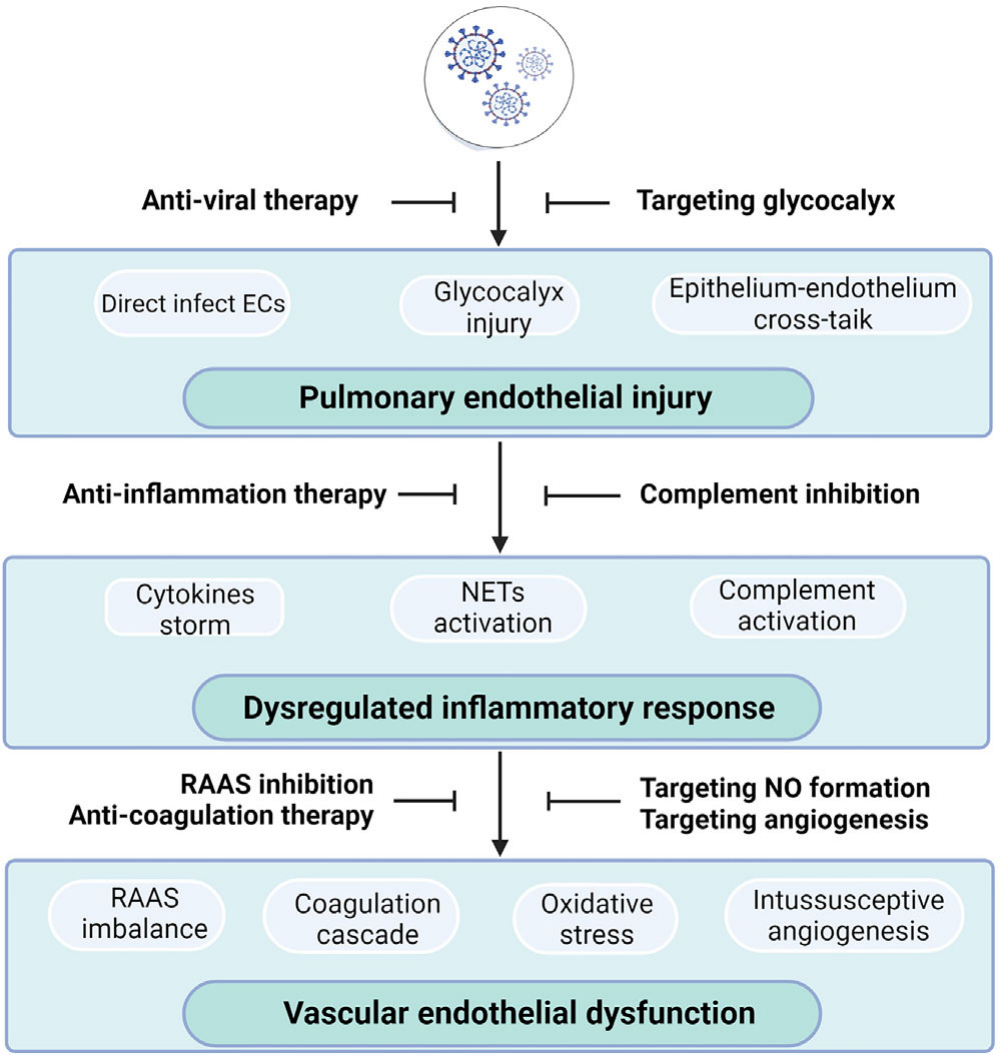
SARS‐CoV‐2 infection‐induced pulmonary vascular injury/dysfunction and potential treatments target the pulmonary vascular system. Pulmonary vascular ECs can be damaged via direct infection of SARS‐CoV‐2, glycocalyx injury, or the crosstalk between the epithelium and endothelium. Thus, anti‐viral therapy is proposed to block SARS‐CoV‐2 entry to prevent damage to ECs, and endothelial glycocalyx is also a potential target. Inflammation cascades could result in cytokines storm, NETs activation, and complement cascade. Therefore, treatment of anti‐inflammation and complement inhibition is urgently needed. Subsequent endothelial dysfunction may be caused by RAAS imbalance, hypercoagulation, oxidative stress, and intussusceptive angiogenesis, which provides therapeutic ideas that target RAAS, nitric oxide production, hypercoagulation, and angiogenesis

An effective treatment strategy for COVID‐19‐associated pulmonary vascular disease is urgently needed owing to the lack of a specific vaccine or available therapies against SARS‐CoV‐2 infection on the pulmonary vasculature. The pathological mechanisms associated with COVID‐19, which disrupts the pulmonary vasculature, may provide novel targets for intervention, and some can be targeted using re‐purposed drugs. Agents that limit pulmonary vascular injury and dysfunction can mitigate RAAS dysregulation, prothrombotic state, excessive inflammation, and angiogenesis induced by SARS‐CoV‐2 infection and can alleviate severe sequelae of COVID‐19 (Figure 4).

### 4.1 Anti‐viral therapy

Antiviral treatment can prevent the occurrence and spread of the SARS‐CoV‐2 infection. During the COVID‐19 outbreak, some potential antiviral drugs were administered to patients with severe COVID‐19. Currently, several monoclonal neutralizing antibodies have been designed for the treatment of COVID‐19 patients.^126^, ^127^ Nucleoside analogs have been widely used as antiviral agents. For instance, Remdesivir, an antiviral prodrug of adenosine analog, initially showed promising outcomes with a 68% clinical improvement in patients with severe COVID‐19 who were treated with the drug.^128^ However, the therapeutic efficacy and safety of Remdesivir should be confirmed through clinical research in patients with COVID‐19. Notably, an antiviral therapy specifically targeting the pulmonary vasculature has not been developed. There is a need to design antiviral drugs or strategies specifically targeting pulmonary blood vessels to protect them from SARS‐CoV‐2 damage.

NO is currently being explored as an experimental treatment for COVID‐19 patients. As an antiviral agent, NO has several advantages. First, it can easily pass through cell membranes to reach adjacent cells and viruses without requiring a receptor.^129^ In addition, NO can be used as a target of several viruses to inhibit viral replication.^130^ Besides, contrary to antiviral lymphocytes, NO effect is independent of immune recognition of infected cells.^129^ Notably, NO is characterized by high reactivity; thus, its antiviral effects may be mediated by reactions with multiple cellular and viral targets, which may be beneficial for host defense, as it may limit the ability of the virus to develop resistance.^82^ Extensive EC injury has been observed in COVID‐19 patients, and thus it is important to explore inducible NOS (iNOS) levels and NO production after SARS‐CoV‐2 infection. Research demonstrates that antiviral effects of NO are attributed to NO donors or by iNOS directly activated by cytokines.^82^ Recent studies used inhaled NO in COVID‐19 patients, and severe hypoxemia indicate that inhaled‐NO therapy has a preventive and/or rescue role owing to its potent vasodilator effect on pulmonary circulation.^131^, ^132^, ^133^

### 4.2 Therapy targeting eGC

Viruses undergo mutation, indicating that the therapeutic strategies against SARS‐CoV‐2 must be versatile. Therapeutic strategies targeting vascular glycocalyx would be effective for patients with both early and severe COVID‐19,^28^ and prevention of eGC damage makes endothelium more stable to defend the host against infection. Therapies targeting vascular glycocalyx can be developed from two aspects: eGC protection and accelerating repair of the damaged vascular glycocalyx. Although several studies report the damage of eGC, no studies have explored pulmonary vascular glycocalyx injury, which serves as a missing link in the intricate pathogenesis of COVID‐19. This indicates that prevention and treatment of pulmonary vascular eGC damage may potentially present positive effects in COVID‐19 patients with vascular dysfunction. In addition, the vascular glycocalyx is fragile, especially in the lungs, and thus its degradation can be an ideal biomarker of endothelial damage for detection and monitoring of COVID‐19.

### 4.3 RAAS inhibition

RAAS plays important roles in COVID‐19; therefore, the effect of RAAS inhibitors including angiotensin‐converting enzyme inhibitors (ACEi) or angiotensin receptor blockers (ARBs) in COVID‐19 is worth studying.^134^ These drugs are currently used for the treatment of cardiovascular diseases and are known to induce expression of ACE2, thus increasing susceptibility to SARS‐CoV‐2. Some studies report that there is no high risk of SARS‐CoV‐2 infection among COVID‐19 patients undergoing ACEi/ARBs therapy. On the contrary, treatment discontinuation presents negative effects to these patients.^135^ Although there is insufficient data on the therapeutic benefit to COVID‐19, the currently prescribed therapies for cardiovascular disease are recommended as RAAS antagonists.^136^ However, their effects on pulmonary vasculature should be further assessed.

A recent study proposed that targeting ACE2 receptor can directly prevent SARS‐CoV‐2 infection by upregulating expression of a disintegrin and metalloprotease 17 (ADAM17).^137^ ADAM17 is a metalloproteinase that participates in shedding of different membrane‐anchored cytokines, cell adhesion molecules, and enzymes such as ACE2.^138^ Increased levels of ADAM17 can accelerate the shedding of ACE2 and reduce the risk of SARS‐CoV‐2 infection. However, ACE2 is an essential enzyme in RAAS, and the downregulation of ACE2 may result in adverse effects. Further studies should accurately explore the benefits and negative effects of the presence and/or activity or absence and/or inhibition of ACE2 on SARS‐COV‐2 infection and thrombosis. Moreover, studies should design novel therapies to restore vascular homeostasis for the treatment of COVID‐19 patients.^137^

### 4.4 Anti‐coagulation therapy

Effective anticoagulation should be ensured owing to the high burden of comorbidities and mortality in COVID‐19 patients with thromboembolic complications. ARDS is the main cause of high mortality in COVID‐19 and is characterized by excessive activity of coagulation cascade, increasing vulnerability to vascular thromboembolic events. Escher et al. reported that a COVID‐19 patient complicated with ARDS improved after inducing effective anticoagulation.^139^ In addition, using anticoagulant therapy with heparin is associated with decreased mortality, mainly in patients with increased D‐dimer,^140^ which is available for the management of COVID‐19 either as a prophylactic or therapeutic regimen. However, a recent study pointed that heparin treatment does not remarkably reduce mortality in severe COVID‐19 cases.^140^ In a recent study, 38.5% of the patients presented with pulmonary embolism even after undergoing anticoagulants prophylaxis, as the hypercoagulability state was mainly attributed to platelets and fibrin.^41^ These findings indicate that anti‐platelet or fibrinolytic drugs should be explored for thrombosis prevention and treatment for COVID‐19 patients in the future.^41^ Antithrombin, a common anticoagulant, is a multifaceted serine protease inhibitor of multiple coagulation factors and protects eGC by binding to heparan sulfate.^141^ Moreover, an emerging review believed that antithrombin is a potential therapy for COVID‐19‐associated coagulation because it can suppress excess inflammation by inhibiting NF‐κB.^142^ Notably, the risk of bleeding should also be considered before the administration of anticoagulation factors in COVID‐19 patients.

### 4.5 Anti‐inflammatory therapy

SARS‐CoV‐2 damages the luminal surface of vasculature and induces vasculitis. Anti‐inflammatory therapies that abrogate cytokine responses should be explored to reduce both morbidity and mortality in COVID‐19 patients.^143^ Several trials are currently underway exploring immunomodulatory therapies, mainly including antagonists of the IL‐1, IL‐6, and JAK/STAT inhibitors.^99^ NETs have a direct role in the immunothrombotic process in COVID‐19; thus, blocking NETs can improve the prognosis of COVID‐19 patients.^144^ In addition, colchicine and some corticosteroids are used for the treatment of COVID‐19‐associated cytokines storm.^16^ However, the effects of anti‐inflammatory agents against COVID‐19 remain debatable. Inflammation is a double‐edged sword as the reaction is a type of body's automatic defense response; however, excessive inflammation is harmful. The untimely intervention of inflammatory regulation is not favorable, and the appropriate drug delivery methods and timing have not been determined. Therefore, further studies should explore a balance to provide a basis for the prevention of cytokine storm associated with COVID‐19.

Previous findings on complement deposition in SARS‐CoV‐2‐damaged pulmonary microcirculation indicate that complement is a potential therapy target for COVID‐19 patients. Complement inhibitors of C3 and C5 have been applied to patients with COVID‐19 and have showed efficacy.^145^, ^146^, ^147^, ^148^ The findings from these studies indicated that complement inhibitors abrogate hyperinflammation in COVID‐19. Transient blocking of C5 can interrupt the hyperinflammatory cycle in COVID‐19 cases, resulting in recovery.^48^ Prolonged complement inhibition, however, is unnecessary for the patient and may increase infection risk.^48^ Besides, studies have also explored an approach to block the MBL pathway by blocking the interaction between MASP‐2 and coronavirus N proteins with anti‐MASP‐2 monoclonal antibodies (nafamostat), which can reduce COVID‐19‐related EC injury and alleviate the risk of inflammation and thrombosis.^149^

### 4.6 Targeting IA

A significant increase in IA is a distinct feature of pulmonary vascular pathobiology, which has been observed through corrosion casting and scanning electron microscopy (SEM) analysis of COVID‐19 lungs and is associated with endothelial dysfunction in COVID‐19 cases. The degree of IA increases with prolonged hospitalization, suggesting that IA is a fundamental mechanism for microvascular expansion and network remodeling; thus, studies should further explore it. Notably, further studies should explore the levels of growth and angiogenic factors such as VEGF, FGF‐2, CXCL12, and CXCR4 in COVID‐19 patients to determine the magnitude of vascular injury after SARS‐CoV‐2 infection. Besides, a loss or detachment of pericytes in alveolar capillaries in patients with COVID‐19 was thought to promote EC sprouting and intussusception,^47^ which provides a novel therapeutic strategy for IA in COVID‐19.

## 5 CONCLUSION

In addition to the known impact on the respiratory system, previous findings indicate that pulmonary vasculature is involved in COVID‐19. Patients with preexisting pulmonary vascular diseases are at a higher risk of morbidity and mortality. EC damage and dysfunction, coagulation cascade, hyperinflammation, and IA are potential pathological mechanisms of pulmonary vascular alteration in COVID‐19. These findings indicate that pulmonary vasculature is a vulnerable target during SARS‐CoV‐2 infection, which is important in pathogenesis and is a potential target for therapy against SARS‐CoV‐2 infection. Further studies should explore the vascular effects of COVID‐19 development and progression to help identify effective therapies and improve the prognosis of patients.

## CONFLICT OF INTEREST

All authors declare no competing interests.

## AUTHORS’ CONTRIBUTIONS

Xiawei Wei contributed to the concepts and revised the manuscript. Jiayuan Ai drafted the manuscript and created the table and figures. Weiqi Hong contributed sectional writing material and helped to revise and polish the manuscript. Min Wu revised the manuscript. All authors read and approved the submitted version.

## ETHICS STATEMENT

Not applicable.

## Data Availability

Not applicable.
